# Can trophectoderm morphology act as a predictor for euploidy?

**DOI:** 10.5935/1518-0557.20180036

**Published:** 2018

**Authors:** Ivan H Yoshida, Monise Santos, Caroline Z Berton, Caroline L Chiarella, Michelli S Tanada, Emerson B Cordts, Waldemar P de Carvalho, Caio P Barbosa

**Affiliations:** 1 Instituto Ideia Fertil de Saúde Reprodutiva, Santo André - SP, Brazil; 2 Faculdade de Medicina do ABC, Santo André - SP, Brazil

**Keywords:** Chromosome, aneuploidy, blastocyst, preimplantation genetic screening

## Abstract

**Objective:**

Euploid embryo transfers yield better implantation rates. In Brazil, morphological evaluation is performed to select the best embryos, since genetic analysis is still an expensive procedure. This study aimed to evaluate whether there is an association between trophectoderm morphology and ploidy status.

**Methods:**

The study included 113 blastocysts formed in D5/D6 from 58 *in vitro* fertilization cycles held from January/2016 to May/2017. All patients with indication for PGD/PGS were included in the study. The mean age of the female patients was 37.04±5.65years. Biopsied blastocysts were categorized for morphology. Cells were sent for genetic analysis using the CGH array, SNP array or NGS techniques. Statistical analysis was performed using the chi square test, and statistical significance was assigned to differences with *p*≤0.05.

**Results:**

Chromosome analysis revealed that 44 (38.9%) blastocysts were euploid. Blastocysts with trophectoderm grades A, B, and C had euploidy rates of 71.43%, 60% and 19.67%, respectively (*p*≤0.05).

**Conclusion:**

Although the best trophectoderm morphology grades had higher euploidy rates, this indicator alone is not enough to warrant embryo genetic viability.

## INTRODUCTION

Infertility is characterized by the inability to reach spontaneous gestation after twelve months attempting to conceive through unprotected intercourse. Approximately 10% of adults of reproductive age have trouble conceiving (ASRM, 2017).

For decades, embryo viability was assessed based on embryo morphology (Ebner *et al*., 2003). Today, embryo genetics is considered a crucial factor in the achievement of healthy pregnancy, since embryos with good morphological scores might be aneuploid (Alfarawati *et al*., 2011). Pre-implantation genetic diagnosis (PGD) and pre-implantation genetic screening (PGS) are of great importance today and have been implemented in most assisted human reproduction clinics. These techniques revolve around genetic tests designed to provide information and help prevent genetic and chromosomal diseases. In these tests, one or more cells have to be harvested from the embryo for analysis (Schoolcraft *et al*., 2010).

The blastocyst is the embryo on the fifth, sixth or seventh day of development, an organism with a differentiated structure and a greater number of cells available for biopsy and genetic analysis in the trophectoderm, the peripheral region of the blastocyst from which the placenta and its annexes originate (Jansen *et al*., 2008). Different molecular testing techniques can be used in embryo cells, including fluorescence in situ hybridization (FISH), Comparative Genomic Hybridization (CGH array or aCGH), Karyomapping and Next Generation Sequencing (NGS). Each is based on a different principle; the choice is made according to the history and needs of each couple.

Microarray analysis with CGH array rapidly gained attention and replaced Fluorescent In Situ Hybridization (FISH), as it enabled the evaluation of ploidy in the 24 chromosomes (Schoolcraft *et al*., 2010). Karyomapping investigates thousands of single nucleotide polymorphisms (SNPs) throughout the genome, allowing the detection of chromosomal abnormalities and the diagnosis of genetic mutations inherited by binding analysis (Handyside *et al*., 2010). Recent developments in NGS introduced improvements in the detection of chromosome aneuploidies when compared to other methods (Handyside, 2013).

This study aimed to evaluate whether there is an association between trophectoderm morphology and ploidy status.

## MATERIAL AND METHODS

### Case series

This retrospective observational study included blastocysts formed on the fifth or sixth day (D5/D6) manipulated in *in vitro* fertilization cycles performed from January 2016 to May 2017 at the Instituto Ideia Fertil de Reprodutiva, in Santo André - Brazil. All patients with indication for PGD and PGS (maternal age ≥37years, >2 miscarriages or >2 implantation failures) were included in the study.

### Laboratory procedures and blastocyst categorization

Controlled ovarian stimulation was performed, and oocytes and semen were collected. The oocytes were denuded three hours after ovarian puncture. Metaphase II oocytes were selected for ICSI. After 16-18 hours, the oocytes were tested for the presence of pro-nuclei. The embryos were cultured in 20µL sequential media: G1 (Vitrolife, Sweden) from D0 to D3, then switched to G2 (Vitrolife, Sweden) from D3 to D6 in humidified incubators with 5% O_2_ and 6.5% CO_2_.

Blastocyst morphology was assessed on D5 and D6. The embryos were categorized based on the procedure published by Gardner & Schoolcraft (1999a,b); they were divided into three groups according to trophectoderm quality: group 1 - Blastocyst 3 to 6A; group 2 - Blastocyst 3 to 6B; and group 3 - Blastocyst 3 to 6C.

The categorization considered the development stage of the blastocysts (expansion and hatching state); score or quality of the internal cellular mass (ICM); and trophectoderm score or quality (TE)

Degree of expansion:

1. The blastocyst cavity occupied less than half the volume of the embryo.
2. The blastocyst cavity occupied more than half the volume of the embryo.
3. Complete blastocyst, with the cavity occupying the entire embryo.
4. Expanded blastocyst, with the cavity larger than the embryo and thinning of the zona pellucida.
5. Blastocyst Hatching
6. Blastocyst hatched

Internal Cell Mass (ICM):

- Many cells, well packed.
- Several cells, loosely grouped.
- Few cells.

Degree of trophectoderm (TE):

- Many cells forming a cohesive layer.
- Few cells, forming a loose epithelium.
- Fewer large cells.

After biopsy, the blastocysts were vitrified and the harvested embryo cells sent for genetic analysis. The method of analysis (CGH array, SNP array or NGS) was chosen based on the examination indications for each couple. Euploid blastocysts were devitrified and transferred in a single embryo transfer cycle.

### Embryo biopsy

On the third day of embryo development (D3), laser-assisted hatching (AH) was performed on the zona pellucida to facilitate the hatching of the cells to be biopsied. Only blastocysts categorized as grade 3 or better were biopsied. During biopsy, six to ten trophectoderm cells were harvested.

All biopsies were performed using a Nikon Ti-S inverted microscope. An OCTAX laser was used in the procedures. The blastocysts were biopsied on plates containing three 10µL drops of Gmopsplus (Vitrolife, Sweden) covered with mineral oil (Irvine Scientific).

### Statistical analysis

Data were treated and statistical analysis was performed using the chi square test. Differences with a *p*<0.05 were deemed significant.

## RESULTS

Biopsies and genetic tests were performed on 113 blastocysts formed in the IVF laboratory from 58 *in vitro* fertilization cycles. The mean age of the female patients was 37.04±5.65 years. The euploid embryo rate was 38.9% (44/113).

Biopsies performed on blastocysts with better trophectoderm morphology were more likely to be chromosomally normal (A and B) when compared to specimens given lower scores (C). Blastocysts with trophectoderm grades A, B, and C had euploidy rates of 71.43%, 60% and 19.67%, respectively (*p*≤0.05), indicating a concomitant drop in morphological quality and euploidy rate.

## DISCUSSION

Embryo selection aims to improve the success rate of assisted reproductive technologies. However, success may be defined in several ways: increased implantation, clinical pregnancy, and live birth rates; decreased miscarriage rates; or absence of chromosomal abnormalities. In other words, an effective selection system may affect success rates in different settings (Macklon *et al*., 2002). One of the most frequently used criteria in the selection of embryos for transfer is morphology. However, embryo morphology studies indicated that selection by this criterion might be imprecise and fail to detect cases of developmental disruption and aneuploidy (Rijnders & Jansen, 1998; Milki *et al*., 2002).

Chromosomal abnormalities are the predominant cause of several clinical problems in natural conception and assisted reproduction contexts (Macklon *et al*., 2002). With the high risk of transmission of chromosomal and genetic mutations and the occurrence of several unsuccessful transfers in mind, PGD has granted patients on assisted reproductive technology protocols the possibility of having euploid embryos transferred for the assessed conditions.

This study investigated whether there is a relationship between the morphological quality of the trophectoderm and the ploidy status of the blastocyst. Our PGD/PGS data revealed a significant association between these parameters, i.e., blastocysts with higher scores were more likely to be euploid. These findings, despite the small size of our population when compared to other published papers, supported the findings described by Fragouli *et al*. (2014), Capalbo *et al*. (2014), and Majumdar *et al*. (2017). Fragouli *etal.* (2014) assessed the morphology of 122 blastocysts on Days 5 and 6, and observed that euploid blastocysts were positively associated to the degree of trophectoderm expansion and quality.

The morphology of trophectoderm cells is extremely important at various times. Honnma *et al*. (2012) compared trophectoderm cells, internal cell mass, and blastocyst expansion for pregnancy and recurrent miscarriage rates. Blastocysts with trophectoderm cells categorized as grades A or B yielded higher pregnancy rates than grade-C trophectoderm cells. Concerning recurrent miscarriage, blastocysts with grade-A trophectoderm cells presented lower miscarriage rates than cells assessed as grade B or C.

Among other things, the study by Majumdar *et al*. (2017) described a correlation between trophectoderm morphology and pregnancy and implantation rates. Blastocysts with trophectoderm cells categorized as grade A yielded higher pregnancy rates, whereas grade-B trophectoderm cells had higher implantation rates. Alfarawati *et al*. (2011) performed a study on blastocyst morphology and aneuploidy. The authors found a statistically significant association (*p*=0.19) between poorer trophectoderm cell grade (C) and higher rates of embryo aneuploidy, as also observed in our study.

A major limitation of studies attempting to find correlations between embryo morphology and ploidy status is that the evaluation of individual morphological parameters may vary significantly because of the subjective nature of visual assessment. The results found in this study indicated that the embryologists in charge of performing morphological categorization did a good job, considering intra-observer variability. Capalbo *et al*. (2014) have demonstrated that biopsied blastocysts of different morphological qualities, if euploid, yielded similar implantation rates. Thompson *et al*. (2013) looked into blastocyst morphology and found that pregnancy and live birth rates were correlated to trophectoderm cell grade, in that higher-grade cells led to higher pregnancy and live birth rates. These parameters were not assessed in this study, since there were few transfers in relation to the number of euploid embryos.

## CONCLUSION

The tools available for embryo selection - including morphological and chromosome evaluation - allow the best embryos to be chosen. The present study demonstrated that although higher trophectoderm morphology scores correlated with higher euploidy rates, this assessment does not replace the need for genetic analysis to reduce the risks of transferring aneuploid embryos.
